# Evaluating the impact of citations of articles based on knowledge flow patterns hidden in the citations

**DOI:** 10.1371/journal.pone.0225276

**Published:** 2019-11-21

**Authors:** Mingyang Wang, Jiaqi Zhang, Shijia Jiao, Tianyu Zhang

**Affiliations:** College of Information and Computer Engineering, Northeast Forestry University, Harbin, People’s Republic of China; National University of Singapore, SINGAPORE

## Abstract

The effective evaluation of the impact of a scholarly article is a significant endeavor; for this reason, it has garnered attention. From the perspective of knowledge flow, this paper extracted various knowledge flow patterns concealed in articles citation counts to describe the citation impact of the articles. First, the intensity characteristic of knowledge flow was investigated to distinguish the different citation vitality of articles. Second, the knowledge diffusion capacity was examined to differentiate the size of the scope of articles’ influences on the academic environment. Finally, the knowledge transfer capacity was discussed to investigate the support degree of articles on the follow-up research. Experimental results show that articles got more citations recently have a higher knowledge flow intensity. The articles have various impacts on the academic environment and have different supporting effects on the follow-up research, representing the differences in their knowledge diffusion and knowledge transfer capabilities. Compared with the single quantitative index of citation frequency, these knowledge flow patterns can carefully explore the citation value of articles. By integrating the three knowledge flow patterns to examine the total citation impact of articles, we found that the articles exhibit distinct value of citation impact even if they were published in the same field, in the same year, and with similar citation frequencies.

## Introduction

The effective evaluation of the impact of a scholarly article is an important research topic, as promotions in the assessment of academia and research grants usually ascribe a significant amount of weight to the impact of an individual’s publication record [1–9].

Researchers have proposed a variety of bibliometrics performance indicators to measure the impact of a single scholarly publication or a set of such publications. These indicators can be classified into two main categories. The first category is citation-based indicators where a citation count becomes the most widely used citation-based index to measure the scientific impact of an article [10–12]. In many academic search engines, such as *Google Scholar*, *Microsoft Academic Search*, and *CiteSeerX*, citation-based analyses have been adopted, and a few online services (e.g., citation count, *h*-index, citation graph) have been provided to users [13]. For publications with the same years and fields, the number of citations is considered to indicate the impact of the articles on the advancement of the fields. To measure the impact of a scientist, a college, and a research institute, the metrics of the *h*-index [14–23], the *g*-index [24, 25], and the *R*-index [26], respectively, have been used. These indexes use the citation count of a publication as the basis for assessing its impact because these are assumed to be valid approaches.

The second category is based on the topological structure of a citation network. The PageRank algorithm is a link analysis algorithm used by Google to rank web pages based on the importance of the web pages. Considering articles as nodes and citations as edges, a citation network is similar to a web graph. Various ranking algorithms based on the topological structure of a citation network were used to assess the impact of a scholarly article in recent years [27–40]. The importance of citing an article, the heterogeneous scholarly network, the publication time, the phenomena of self-citation, and missing citations are usually considered for these studies. Different weights are given to the edges in the citation network when considering the aforementioned factors to evaluate different citation relationships among articles.

While the aforementioned researches seem to suggest that citations may exhibit different levels of importance, they do not explicitly reveal what these levels are and how to detect and use these different levels to evaluate the impact of an article. In one of our previous work, we made a preliminary discussion on the detection of various hidden patterns in citations [41]. The indices of citation intensity, citation width, and citation depth were proposed to distinguish unequal intensities and contributions in citations [41]. In fact, the occurrence of a citation will be accompanied by knowledge flow. Knowledge will be propagated from the cited article to the citing one when the citation activity occurs [42]. Articles will show different knowledge flow patterns even if they have the same number of citations. First, articles will have different knowledge flow intensity. Since the occurrence of citation behavior can represent the knowledge flow behavior between articles, the intensity of knowledge flow in articles can be measured by whether they have a strong ability to be continuously cited. This actually reflects whether an article has strong vitality to get more citation opportunities. Obviously, articles with strong citation vitality will have high academic influence. Therefore, in determining the citation impact of articles, we should consider the intensity of knowledge flow of articles. Second, articles will have different knowledge diffusion capabilities. Each academic article comprises multiple attributes, such as publishing journals, subjects, authors’ organizations, and countries. When citations occur, knowledge diffusion among articles already has been completed, from the attribute space represented by the cited articles to the attribute space of the citing articles. If an article can be cited by larger number of different countries, institutions, disciplines, journals, and others, it shows that the knowledge of the article has an impact on the scientific research of more academic entities. This impact reflects the scope of the influence of the knowledge of the cited article on the academic environment through the occurrence of citations. When the citation impact of an article is evaluated, it is obvious that the knowledge diffusion ability of the article should be considered. Finally, articles will have different knowledge transfer capabilities. The knowledge transfer capability of an article is defined by the extent the article provides support to scientific research based on the occurrence of citations. It can be assumed that if an article can be cited with more high-quality academic achievements and has larger content similarities with these academic achievements, it can be considered that the article has a high ability to transfer knowledge. We should consider the capacity of knowledge transfer when measuring the citation impact of an article.

Therefore, in this paper, we extract the three knowledge flow patterns mentioned above to evaluate the citation impact of an article. The remainder of this paper is organized as follows: The “Methodology” section gives the detailed process of measuring the academic impact by using the knowledge flow patterns. This is followed by the “Data” section, which deals with the data used in the experiments. In the “Experimental results and discussion” section, the experimental results are presented. The final section with a summary of the overall discussion concludes the paper.

## Methodology

### Knowledge flow intensity

The intensity of knowledge flow reflects whether articles have larger vitality to obtain more citations, which cannot be effectively obtained by analyzing the total frequency of citations. One of our previous studies discussed about the articles that have a stronger ability to be cited consistently [43]. By developing a series of time windows, we have explored the correlation between citation frequency under different time windows and future citation ability of articles and found that neither highly cited articles nor newly published articles can absolutely have stronger future citation ability. Articles with strong sustained citation ability are those that have been cited more frequently over the past two years, regardless of their total citation count and publication date [43]. In this paper, we have considered a similar idea to construct independent and continuous time windows to explore the knowledge flow intensity of articles in different time periods and thus determine which articles should have the larger knowledge flow intensity at present.

1. Independent time windows: Taking a year as a unit, a series of independent time windows was established. A time parameter *T* is used to represent the size of the independent time window. *T = 1* denotes 2015; *T = 2* denotes 2014. The rest are calculated in the same manner.
2. Continuous time windows: Taking a year as a unit, we established a series of continuous time windows, gradually increasing by 1 year. A time parameter *τ* is used to represent the size of the continuous time window. *τ = 1* denotes 2015 (the previous year); *τ = 2* denotes the recent 2 years—2015 and 2014. The rest are calculated in the same manner.

According to the above methods of dividing independent and continuous time windows, this paper bifurcated the total citation frequencies of articles into the citation frequencies obtained in different time windows. Moreover, the citation counts of articles obtained in 2016 were used to represent the ability of the articles to be continuously cited in the future. By exploring the correlation between citation frequencies under different time windows and future citation ability of articles, this paper aims to distinguish the various abilities of citations obtained under different time windows to represent the sustained citation behavior of articles. Pearson’s correlation coefficient is used to define this relationship, which is calculated as follows:

$$r_{i}=\frac{\sum_{j}\left(X_{i}{}_{j}-\bar{X_{i}})(Y_{j}-\bar{Y}\right)}{\sqrt{\sum_{j}{\left(X_{ij}-\bar{X_{i}}\right)}^{2}}\times \sqrt{\sum_{j}{\left(Y_{j}-\bar{Y}\right)}^{2}}},$$ (1)

where *r*_*i*_ is the Pearson’s correlation coefficient between the articles’ future citation counts and their past citations obtained in the *i*th time window. *X*_*ij*_ denotes the past citations in the *i*th time window for article *j*. ${\bar{X}}_{i}$ is the average of citations in the *i*th time window for all articles. *Y*_*j*_ denotes the future citation counts of article *j* obtained in 2016. $\bar{Y}$ is the average of future citation counts of all articles obtained in 2016.

The correlation coefficient calculated under different time windows can be used to explore the contribution of citation frequencies in different time windows to help articles attract new citations. The larger the correlation coefficient is, the higher the contribution of citations to the future under this time window. When determining the overall knowledge flow intensity of an article, citation frequencies obtained by the article in this time window should be given a higher weight. This paper considers correlation coefficients as the weight of citation frequencies obtained under corresponding time windows. Based on the weighted accumulation of the citation frequencies under each time window, the total knowledge flow intensity of the article can be obtained. This reflects the influence of citations with regard to knowledge flow intensity.

### Knowledge diffusion capacity

Citation activities should not be expressed solely as numbers, but they should be reflected as a wide space distribution pattern from the perspective of knowledge diffusion. Every article should be seen as a carrier of knowledge, and every citation activity between articles contains the diffusion process of knowledge from the cited article to the citing one. In the earlier work done by the authors to detect the typical features influencing the citation impact of an article, we found that a wider citation distribution in various subjects, journals, countries, and institutions had a greater influence on increasing the citation impact of articles [41, 44–45]. Thus, this work constructed the feature space *F* from the aforementioned four features to describe the knowledge diffusion process among articles:

F={SubjectCategory,Journal,Country,Institution}

Detecting citation distribution patterns of cited articles on this feature space can provide important information about the size of the scope of their influence on the scientific environment. A wider citation distribution in feature space *F* for one article, i.e., a larger scope of influence, would be more advantageous in the knowledge diffusion of the article.

To analyze the knowledge diffusion capacity of articles, the theory of mutual information is introduced to calculate the dependency of the total citation counts of articles on each of the feature dimensions in *F*. Mutual information is a statistical measure of interactions among variables and can linearly/nonlinearly access their dependency [46–47]. The mutual information between variables *X*_*i*_ and *Y* is defined by the following equation:

$$I(X_{i},Y)=\sum_{Y\in R}\sum_{X_{i\in F}}p(X_{i},Y)\log_{2}\frac{p(X_{i},Y)}{p(X_{i})p(Y)},$$ (2)

where *X*_*i*_ denotes the citation distribution in the *i*th dimension of feature space *F* and *Y* denotes the total citations of articles. *p(X*_*i*_*)* and *p(Y)* are probability density functions, and *p(X*_*i*_,*Y)* represents the joint probability function. Mutual information is a nonnegative concept, i.e., *0≤I(X*_*i*_*;Y)≤1*; the value *I* = 1 indicates the highest dependency, and 0 denotes no intercorrelation. The dependency provides important information to analyze the contribution of knowledge diffusion properties of articles to their total citations in each feature dimension; the dependency is also the weight of the number of citations in this dimension. Then, the knowledge diffusion capacity of each article is quantified by the accumulation of weighted citations from each feature dimension in *F*.

### Knowledge transfer capacity

The knowledge transfer capacity can be used to represent the extent to which the cited article supported subsequent research. Based on the number of citations, it is hard to analyze the extent of support for citing articles from cited articles. This paper presented a method based on deep learning technique to calculate the similarities in the content of cited articles and highly cited citing articles to quantify the support of cited articles to the subsequent research.

Deep learning has been successful in several fields because of the strong ability of feature learning and modeling [48–49]. The use of distributed representation in deep learning has shown high effectiveness in capturing the semantics of words, phrases, and sentences, which benefits natural language–processing applications such as sentiment analysis [50], syntactic parsing [51–54], text summarization [55], and others [56–59]. This research has explored the use of distributed document representation in calculating the content similarity between articles. We proposed to use the Doc2vec method, which builds a distributed vector representation at the document level using an unsupervised approach [60].

To achieve the knowledge transfer capacities of one article, it is time-consuming and unnecessary to collect all the citing articles to it. Because highly cited papers (HCPs) could represent high-quality research findings in a less rigorous manner, highly cited citing papers (HCCPs) were extracted from an article to examine the number of high-quality research findings generated under the support of an article. Then, we calculated the similarity between the content of the article and its HCCPs to evaluate the knowledge transfer capacity of the article. This was done based on the condition that if article *A* was cited by more HCCPs and *A* had a larger content similarity with these HCCPs, it could confirm that article *A* had more knowledge transfer capabilities in the subsequent studies, compared to articles that did not have much HCCPs and enough similarities with HCCPs either.

Suppose, there are *N* papers in the corpus comprising all cited articles and HCCPs citing to them, and we want to learn the distributed document vector such that each paper is mapped to a fixed dimension. There are two models of the Doc2vec method: Distributed Memory Model of Paragraph Vectors (PV-DM) and Distributed Bag of Words version of Paragraph Vector (PV-DBOW) [60]. In our experiment, each document vector is a combination of these two vectors: one learned by the PV-DM and one learned by the PV-DBOW, which are the same in Le & Mikolov's work [60]. The learned document vector representations have 50 dimensions in both PV-DM and PV-DBOW; This means that each paper is mapped to a distribution vector with 100 dimensions.

Suppose, *p*_*i*_ and *Hc*_*ij*_ denote the document vector representations of the *i*th article and the *j*th paper in HCCPs to *p*_*i*_. We calculated the content similarity between the *i*th article and the *j*th HCCPs citing to it with a cosine similarity:

$$c(i,j)=\frac{p_{i}•Hc_{ij}}{\left\|p_{i}\right\|\left\|Hc_{ij}\right\|}$$ (3)

For the *i*th article, the knowledge transfer capacity is calculated as the accumulation of *c(i*,*j)* (*j = 1*,*2*, …*m*), where *m* is the number of HCCPs citing to it.

Obviously, one article can be regarded as having a high supporting value for follow-up research if it has more number of HCCPs and the larger content similarity with these HCCPs as well.

### Evaluating articles’ citation impact from the above three knowledge flow patterns

After quantifying the three knowledge flow patterns mentioned above, the most important question is how to integrate these three patterns to make a holistic assessment of articles’ citation impact? In this paper, the entropy weight method was used to weigh these three patterns and achieve a universal analysis on articles’ citation impact. The method is considered as an objective method for weight calculation because weighting factors are dependent on the value of indices rather than on human subjective assessment [61]. It is derived from Shannon entropy, which was first proposed as a quantitative measurement of uncertainty in the information system. Main steps involved in this process are:

**Step 1:** Initialization of the matrix. Assuming that there are *m* articles that need to be evaluated in terms of *n* indices. In this paper, *n = 3* refers to the three knowledge flow patterns mentioned above. The initial matrix is established as follows:

$$A={\left(a_{ij}\right)}_{m\times n}=\left[\begin{array}{ccc} a_{11} & \cdot \cdot \cdot & a_{1n}\\ \vdots & \ddots & \vdots \\ a_{m1} & \cdots & a_{mn} \end{array}\right].$$ (4)

**Step 2:** Normalization of the matrix. To solve the uniformity of indices’ units or a value range, the normalization of all indices is performed as

$$r_{ij}=\frac{a_{ij}-\min_{j}(a_{ij})}{\max_{j}(a_{ij})-\min_{j}(a_{ij})}(\max_{new}-\min_{new})+\min_{new},$$ (5)

where [min_new_, max_new_] is the new value range for all the indices, which is set as [min_new_, max_new_] = [0.001,0.999].

**Step 3:** Calculation of the weighting coefficient. The information entropy of each index is calculated by

$$E_{j}=-{\left(\ln_{m}\right)}^{-1}\sum_{i=1}^{m}p_{ij}\ln p_{ij},$$ (6)

where *E*_*j*_ is the information entropy of each index and pij=rij∑i=1mrij.

Based on the value of information entropy *E*_*j*_, the weighting coefficient of each index is calculated by

$$w_{j}=\frac{1-E_{j}}{\sum_{j=1}^{n}\left(1-E_{j}\right)}=\frac{1-E_{j}}{n-\sum_{j=1}^{n}E_{j}},$$ (7)

where $\sum_{j=1}^{n}w_{j}=1$ and 0≤*w*_*j*_≤1. *1-E*_*j*_ indicates the inconsistency degree of each article under the *j*th index from the theory of information entropy. Then, the index that can create a larger inconsistent degree among articles, or has a larger capacity to discriminate articles, will have a larger weighting coefficient.

**Step 4:** Calculation of the universal evaluation value on articles’ citation impact:

$$CI_{i}=\sum_{j=1}^{n}w_{j}a_{ij}$$ (8)

Following the steps discussed above, each article can get its universal citation impact *CI*_*i*_ by integrating the three knowledge flow patterns.

## Data

In this paper, two data sets are used to verify the above methods. Data set 1 is mainly considered to analyze the three knowledge flow patterns. Data set 2 is performed to complete the evaluation of universal citation impact on articles.

### Data set 1

We selected the field of “Astronomy and Astrophysics” for our experiments because its publications are well covered by journal publication databases [62] and because it is widely separated from other fields [63]. There are 62 journals in the field of “Astronomy and Astrophysics” in the *Journal of Citation Reports (JCR) 2015* of the *Science Citation Index* (SCI). Among these journals, only 28 have published articles in 1985. We collected 7408 articles from these 28 journals and their citation data during 1985–2016. These articles will test the three knowledge flow patterns proposed in this paper. The detailed information of the 28 journals and the number of articles collected from each journal are listed in Table 1. This data set will be used to discuss the three knowledge flow patterns hidden behind articles’ citation activities.

**Table 1:** Journals and their articles used for our experiments.

No.	Journals	Articles	HCPs	MCPs	LCPs
1	Annual Review of Astronomy and Astrophysics	13	3	8	2
2	Astrophysical Journal Supplement Series	92	10	57	25
3	Annual Review of Earth and Planetary Sciences	16	0	11	5
4	Space Science Reviews	141	0	14	127
5	Astrophysical Journal	1207	22	531	654
6	Astrophysical Journal Letters	3	1	2	0
7	Astronomy and Astrophysics	779	1	174	604
8	Monthly Notices of the Royal Astronomical Society	465	3	156	306
9	Physics Letters B	1520	17	357	1146
10	Astronomical Journal	271	3	76	192
11	Physical Review D	944	10	195	739
12	Publications of the Astronomical Society of the Pacific	240	1	26	213
13	Astroparticle Physics	1	0	1	0
14	ICARUS	160	1	51	108
15	Solar Physics	199	1	35	163
16	Classical and Quantum Gravity	107	0	19	88
17	Revista Mexicana de Astronomia y Astrofisica	77	0	0	77
18	Publications of the Astronomical Society of Japan	56	1	7	48
19	Planetary and Space Science	142	0	17	125
20	Annales Geophysicae	92	1	11	80
21	Astrophysics and Space Science	385	0	7	378
22	General Relativity and Gravitation	97	0	5	92
23	Celestial Mechanics & Dynamical Astronomy	1	0	0	1
24	Radio Science	162	1	25	136
25	Astronomische Nachrichten	48	0	0	48
26	Geophysical and Astrophysical Fluid Dynamics	52	0	6	46
27	Earth Moon and Planets	32	0	0	32
28	Journal of Astrophysics and Astronomy	26	0	1	25
Total	--	7408	76	1792	5540

To investigate the intensity properties of knowledge flow, the citation distribution data of them were divided into two time periods: 1) The first time period is across the time interval from each article’s publication year until 2015. The citation data collected in this time period were used to model the articles’ citation behavior in different time windows. 2) The second time period was established by the citation data of 2016. This data set was used to determine the sustained citation ability of articles in the near future.

When analyzing the knowledge diffusion capacity of each article, the citation distribution data in feature space *F = (Subject Category*, *Journal*, *Country*, *Institution)* in its citing environment were gathered. Based on the “Analyze Results” tool provided by the web version of *SCI*, these data were collected to evaluate the article’s knowledge diffusion capacity.

As for the knowledge transfer capacity of one article, the HCCPs were selected based on the selection method that a citing paper would be of high quality if the number of citations it received was at least ten times the mean citation rate among all citing articles. The selection method was similar to the one applied by Aksnes [64] in the study of HCPs by Norwegian authors. There were 237,458 citing articles with 11,707,971 citations. The average citation rate of these citing articles was 49.305. Thus, the citing articles that had been cited at least 493 times were taken as the HCCPs. Then, all the cited articles and their HCCPs were downloaded, which would be transformed into distributed vector representations by the Doc2vec method to facilitate the calculation on content similarity.

To better understand the different capabilities of articles in knowledge diffusion and knowledge transfer, we have segmented the collected documents into three categories according to their total citations up to the statistical year.

1. HCPs: The HCPs in the field of “Astronomy and Astrophysics” are selected by using the same criterion that was used for the HCCPs. Moreover, a threshold of ten times is used in the selection process.
2. Medium-cited papers (MCPs): An article is considered as medium cited if the number of citations received is in the range of 1–10 times the mean citation rate in the field of “Astronomy and Astrophysics.”
3. Low-cited papers (LCPs): This includes the rest of the publications that received a less number of citations than the mean citation rate.

Table 1 lists the number of articles classified into HCPs, MCPs, and LCPs of each journal.

It should be mentioned that we chose 10 times of the average cited frequency as the standard to select HCPs, which has nothing to do with the subject area, mainly to ensure that an appropriate number of articles are selected as HCPs. According to this standard, only 76 of the 7408 articles published in the field of *Astronomy and Astrophysics* in 1985 were selected as HCPs, accounting for 1% of the total number of papers published in that year, which is consistent with the standard of selecting HCPs in *Web of Science*. In *Web of Science*, papers received enough citations to place it in the top 1% in the same subject area and in the same publication year are classified as highly-cited papers.

### Data set 2

One of the key journals in the field of “Astronomy and Astrophysics,” i.e., *Astrophysical Journal*, was used in the experiment on examining articles’ citation impact. Four highly cited articles published in 1985 in *Astrophysical Journal* were extracted and used to compare their universal citation impact by considering the knowledge flow patterns. These four articles were divided into two pairs, and the total number of citations of the same pair of articles is the same. We choose papers from the same field, published in the same year and with the same cited frequency for analysis, so as to clearly reveal that there are great differences in the citation impact of these papers that meet the same impact standard in traditional citation analysis. The selection method is similar to Yu et al.’s work [65], they chose four papers with similar publication time and the same total number of citations to compare the citation characteristics of them. All the citation data required for detection were collected as discussed in “Data set 1.” The detailed information of these four articles is listed in Table 2.

**Table 2:** Detailed information about four articles.

ID.	Title	First author	Number of citations
HCP-1	Revival of a stalled supernova shock by neutrino heating	Bethe, H.A.	557
HCP-2	The spectra of narrow-line seyfert-1 galaxies	Osterbrock, D.E.	557
HCP-3	Massachusetts stony-brook galactic plane co survey-the galactic disk rotation curve	Clemens, D.P.	478
HCP-4	Thermal infrared and nonthermal radio - Remarkable correlation in disks of galaxies	Helou, G.	478

## Experimental results and discussion

### Experimental results for the intensity characteristics of knowledge flow

**Fig 1:**
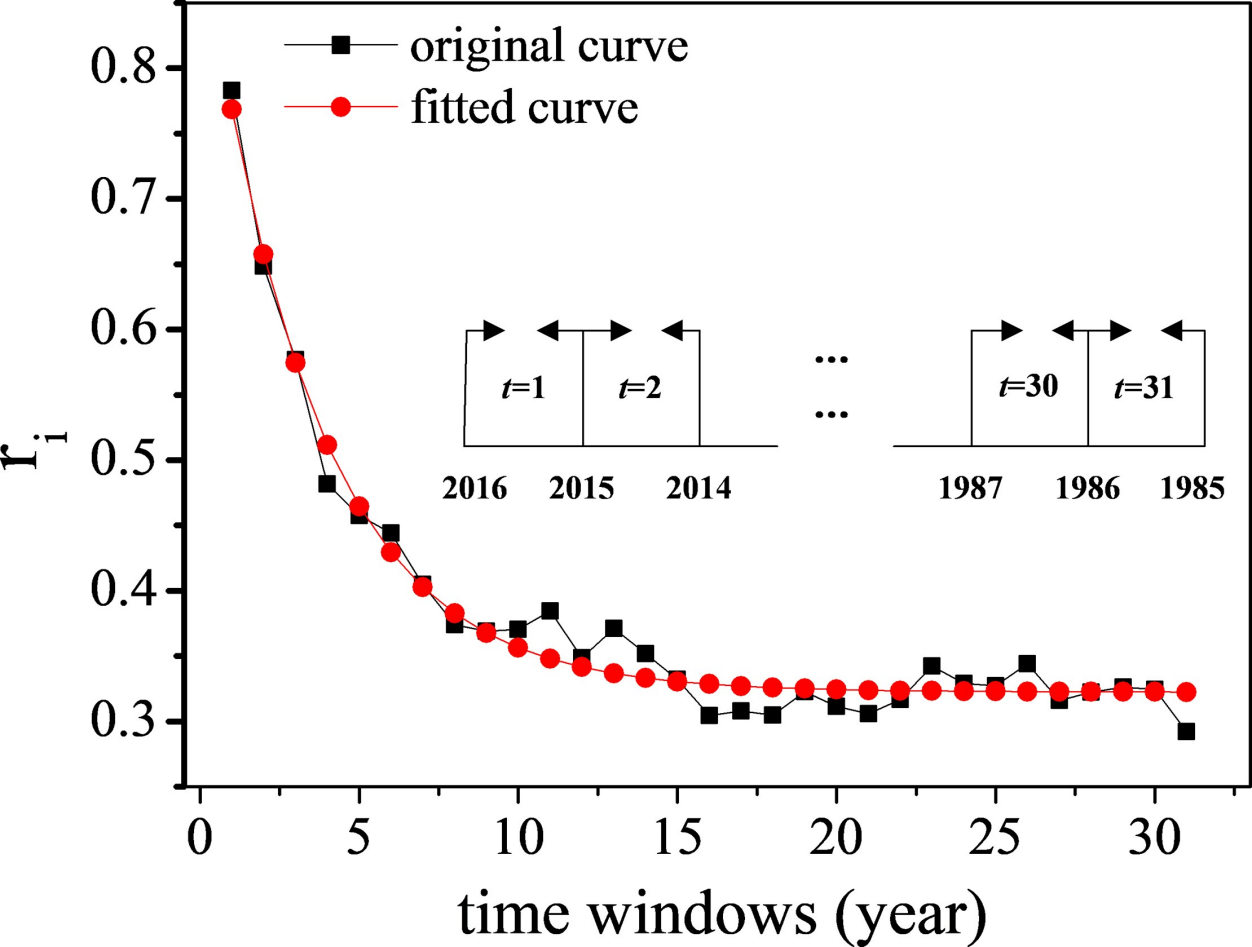
Correlation between articles’ future citation performance on their past citations obtained in different independent time windows.

**Fig 2:**
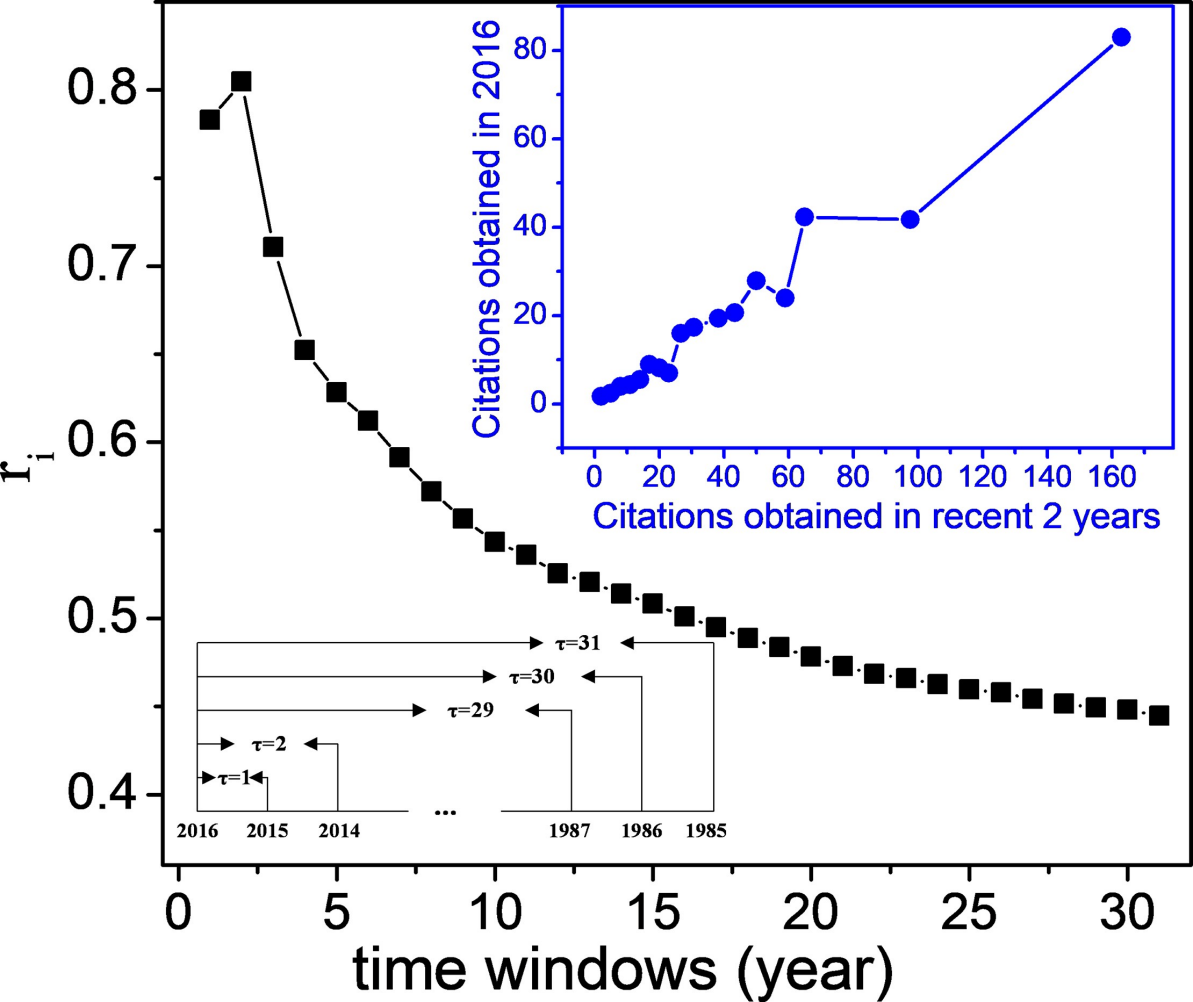
Correlation between articles’ future citation performance on their past citations obtained in different increasing time windows.

Figs 1 and 2 show the correlation between articles’ sustained citation performance and their citation frequency under independent and continuous time windows, respectively, and the subgraph depicts the division of the time windows. The articles’ citations obtained in different time windows generate various influences on their future citation possibilities. The correlation coefficient *r*_*i*_ in Fig 1 decreases continually and exponentially along with the time. The result indicates that the citations obtained in recent years have more impact on articles’ future citation performance. Fig 2 shows time periods in which the citations have the greatest influence on the future citation behavior of the articles. The correlation coefficient in Fig 2 achieves its peak when *τ* = 2, and then it decreases exponentially with the enlargement of the time window, showing that the citations in the past 2 years have more impact on articles’ future citation performance. The subgraph in the upper right corner in Fig 2 depicts the dependence of articles’ future citations on the number of citations obtained in the past 2 years. There is a clear linear dependence, which suggests that the articles obtaining a large number of citations in the past 2 years have a greater chance of being cited again in the near future.

Figs 1 and 2 show different knowledge flow intensities of articles in different time periods. The articles with a large number of citations in recent years will have more knowledge flow intensities to attract new citations. When evaluating articles’ universal citation impact, these different presentations on knowledge flow intensity, representing as diverse correlation coefficients in different time windows, will be used to weigh the past citations to achieve an integral value for the knowledge flow intensity of one article.

### Experimental results for knowledge diffusion capacities

**Fig 3:**
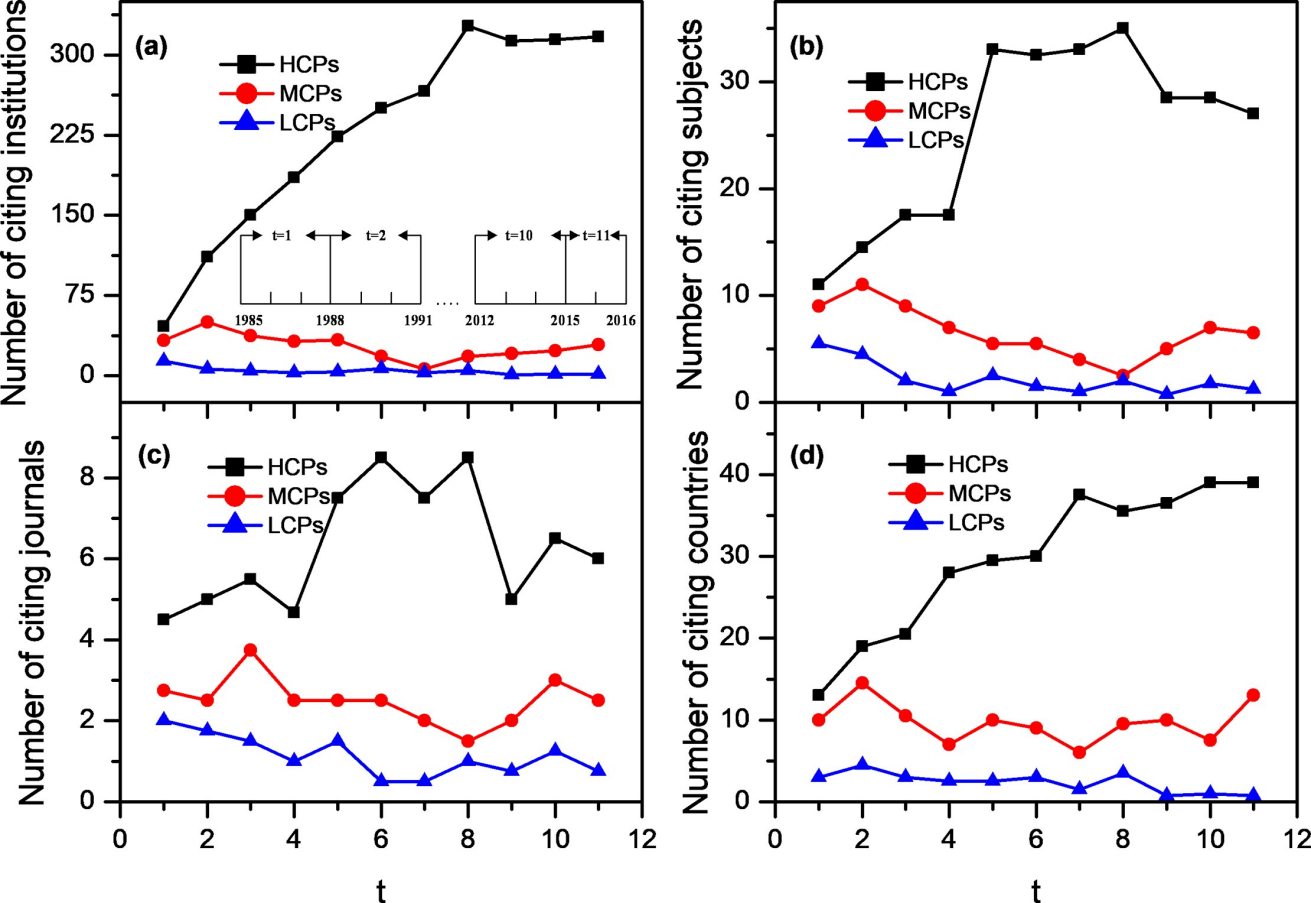
The citation distribution of three kinds of articles in the four features of institutions (a), subject categories (b), journals (c) and countries (d).

Fig 3(A)–3(D) shows the citation distribution of three types of articles (HCPs, MCPs, and LCPs) based on institutions, subject categories, journals, and countries, respectively. To better understand the characteristics of knowledge diffusion of articles, this paper explored the evolution of knowledge diffusion ability of different types of articles in time series. The subgraph in Fig 3(A) shows the classification of time windows. The three types of articles showed a distinct distribution based on each of the four features. The HCPs achieved most citations from institutions, subject categories, journals, and countries in each time window. The MCPs came in second behind the HCPs, while the LCPs acquired the lowest number of citations. Therefore, articles will have a diverse scope of their influences on the academic environment, although they were the same initially when published in the same field and in the same year. Those papers that eventually grew up to be highly cited exhibited the best knowledge dissemination performance in the early post-publication period and at other stages of the whole life cycle. Such a dominated citation distribution in feature space *F* produces better visibility effects for these papers, suggesting that more attention from a wider range of fields can bring more citations in the later years and which has further facilitated the articles becoming highly cited eventually. As a result, the range of articles’ influences on the academic environment also indicates the diffusion characteristics of articles’ citation impact, which is incorporated as one dimension in our scheme to calculate the universal citation impact of articles.

### Experimental results for knowledge transfer capacities

Table 3 shows the results of the indicators used for evaluating articles’ knowledge transfer capacities. Obviously, the average number of HCCPs for the HCPs is much bigger than that for the MCPs and LCPs. There are more high-quality research outputs generated in the citing environments of the HCPs. Undoubtedly, those high-quality outputs are probably not directly dependent on one HCP, and there are various reasons for citing one article. However, a large number of high-quality research outputs in the citing environment can be an indicator, to some extent, for demonstrating the high citation impact of the HCPs on subsequent research.

**Table 3:** Indicators to evaluate knowledge transfer capacities for three kinds of articles.

Cited articles	Average number of HCCPs	Average content similarities
HCPs	5.91	0.327
MCPs	0.50	0.079
LCPs	0.08	0.01

The last column in Table 3 lists the average content similarities between different kinds of cited articles and their HCCPs. As shown in Table 3, the average content similarity for HCPs is 0.327, which is more than MCPs (0.079) and LCPs (0.01). It indicates that HCPs have the highest content similarities with high-quality research outputs, besides the dominated average number of HCCPs.

Therefore, articles will show completely different characteristics of knowledge flow through the occurrence of citation behavior, which cannot be detected only by the characteristics of the number of citations. These knowledge flow patterns have not only shown the diverse intensity, scope, and depth of articles’ influences, but have also benefited generating diverse citation life of them. In the next experiment, we have incorporated articles’ knowledge flow patterns into the evaluation of the citation impact and verified the necessity of doing so using the article data in “Data set 2.”

### Experimental results for evaluating articles’ citation impact

The citation impact of the two pairs of highly cited articles in Table 2 was universally examined by integrating the knowledge flow patterns.

As for the intensity characteristic of knowledge flow, the citation frequency obtained in the independent time window of *T = i* were weighted by the correlation coefficient *r*_*i*_ in the same time period. Then the weighted citations in different independent time windows were accumulated to quantify articles’ total intensity characters of knowledge flow. As for the knowledge diffusion capacities, mutual information was used to calculate the dependency of the paper’s total citation counts on each of the feature dimension in *F*, which is the weight for each feature dimension. Then, the weighted citations from each feature dimension were accumulated to quantify the knowledge diffusion capacity of each article. As for the knowledge transfer capacities, we collected the HCCPs for each article in Table 2, and used the deep learning model of Doc2Vec to calculate the content similarity between each article in Table 2 and its corresponding HCCPs. After quantifying the knowledge flow patterns, the entropy weight method was used to weigh each pattern and calculate the universal citation impact of each article.

Table 4 lists the citation distribution data of the four articles in each feature dimension, as well as the final quantified knowledge diffusion capacity of them. The results show that the knowledge diffusion ability and the overall knowledge diffusion performance of the articles are completely different in each dimension of *F*, even if they have the same or similar citation counts. The diverse knowledge diffusion capacities show the diverse influences of the articles on the academic environment.

**Table 4:** Knowledge diffusion capacities for four articles.

ID	Number of citing subjects	Number of citing journals	Number of citing countries	Number of citing institutions	knowledge diffusion capacity
HCP-1	18	139	47	429	112.34
HCP-2	22	92	43	439	99.01
HCP-3	13	73	45	555	105.044
HCP-4	9	55	46	562	99.578

**Fig 4:**
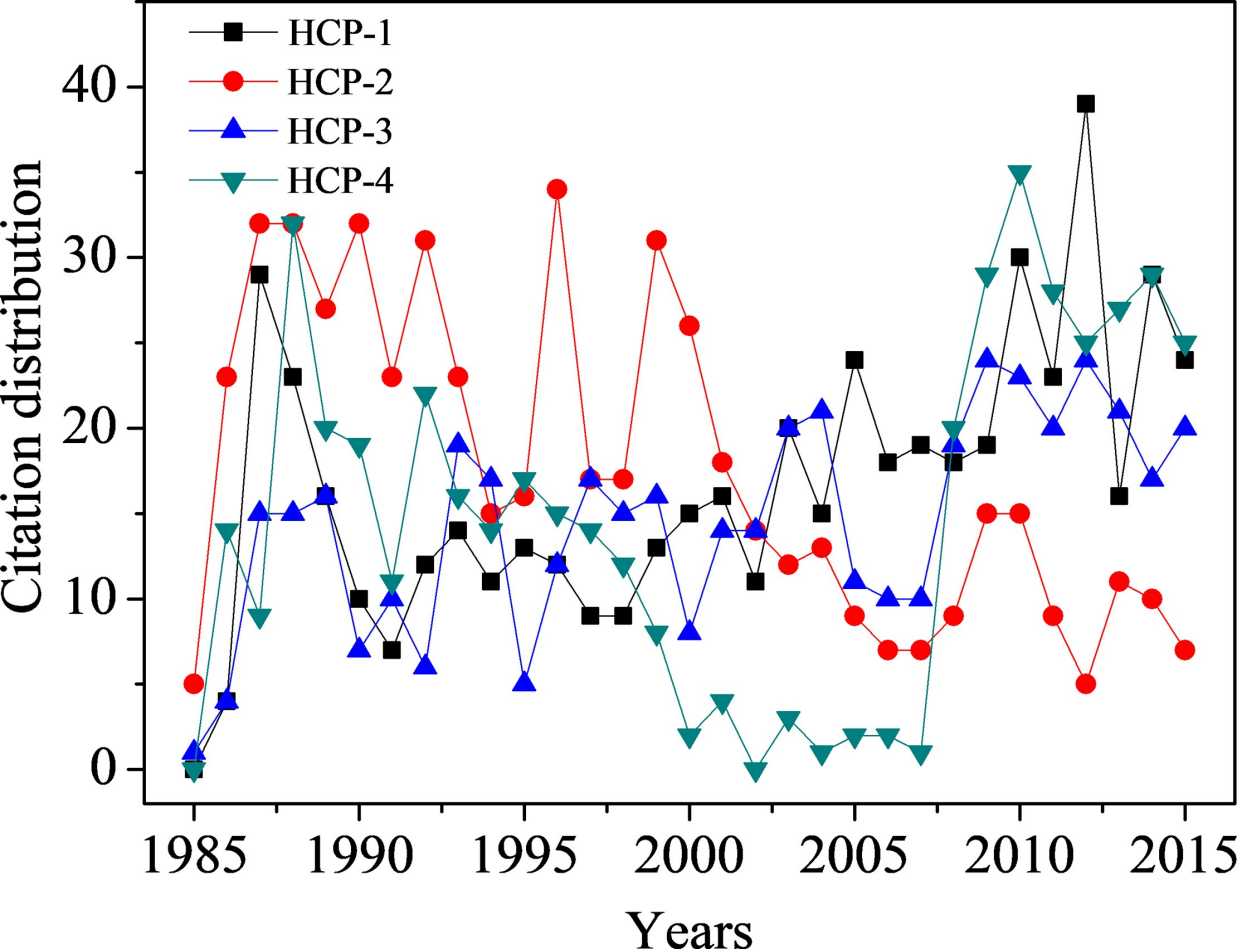
The citation distribution of four articles from their publication year 1985 to 2015.

Table 5 shows the experimental results of the universal citation impact of the articles. The articles in the same pair have shown different universal citation impact after the integration of the information from the knowledge flow patterns, especially in the first pair, HCP-1 and HCP-2. HCP-1 shows a more significant citation impact than HCP-2 because HCP-1 exhibits better than HCP-2 in all three aspects of knowledge flow. At the same time, HCP-4 has shown a little higher citation impact than HCP- 2, although HCP-4 has a much lower citation count than HCP-2. There are eight HCCPs in the citing environment of HCP-4, far more than the three HCCPs in HCP-2’s citing environment. It benefits HCP-4 a better performance in knowledge transfer capacity than HCP-2. In the other two knowledge flow patterns, two articles almost matched. However, we still want to detect their citation distributions to observe their citation performance in recent years. Fig 4 shows the articles’ citation distribution from 1985 to 2015. It is easy to find that the three articles of HCP-1, HCP-3, and HCP-4 show strong citation vitalities in recent years, especially for HCP-4, which exhibits a vigorous citation performance from 2008. At the same time, HCP-2 lacks motivation. As shown in the subgraph in Fig 2, articles that got more citations in recent years will have higher knowledge flow intensity to attract more citations in the future. Therefore, HCP-4 is more active than HCP-2 in attracting new citations.

**Table 5:** Universal citation impact for the four articles.

ID	knowledge flow intensity capacity (weight:0.262)	knowledge diffusion capacity (weight:0.402)	knowledge transfer capacity (weight:0.336)	Integrated citation impact *CI*_*i*_	Number of citations
HCP-1	211.734	112.34	2.338	101.4784	557
HCP-2	192.645	99.01	0.9501	90.6482	557
HCP-3	181.091	105.044	1.0776	90.0819	478
HCP-4	190.757	99.578	2.404	90.8691	478

Thus, it can be concluded that articles will show completely diverse citation impact, even if they have the same or a similar number of total citation counts. Moreover, articles can show similar citation impact, even if they have diverse citations. The results indicate that it is necessary to make this analysis by incorporating the different knowledge flow patterns hidden behind articles’ citation activities.

## Conclusions

In this paper, different knowledge flow patterns hidden behind the number of citations were identified to describe the impact of articles. The purpose of this paper was to highlight that the citation count of articles should not only be seen as a single number, but consideration should also be given to the process of knowledge flow hidden behind the number of citations. According to the idea being put forward, three knowledge flow patterns—the intensity properties of knowledge flow, the knowledge diffusion capacities, and the knowledge transfer capacities were identified and incorporated to examine the citation impact of an article. This paper collected articles related to the field of “Astronomy and Astrophysics” to analyze the performance of them in the knowledge flow patterns, and accordingly discussed the necessity of incorporating these three patterns into citation impact assessments. Experimental results showed that articles present diverse knowledge flow intensities in different time windows. Those having higher citation counts in recent years have large knowledge flow intensities to be cited more in the future. HCPs received more citations in the four feature dimensions of knowledge diffusion in all the time periods. A broader distribution under these features would be a considerable proof for the large scope of their influence in the academic environment, as well as evidence for more visibility in the generation of more citations in the future. HCPs had a greater possibility to be cited by more high-quality research outputs. Considering major content similarities of HCPs with these research outputs, HCPs are more active in knowledge transfer. Thus, articles have shown diverse intensity, diffusion, and transfer characteristics of knowledge flow. These different representations regarding the characteristics of knowledge flow contribute to the formation of different citation trajectories of articles, even if they were published in the same subject area and in the same year. It is necessary to incorporate the three patterns when making a universal analysis on articles’ citation impact.

To detect whether articles with the same citation counts would still have the same citation impact after considering the three knowledge flow patterns, four highly cited articles in *Astrophysical Journal* were collected and divided into two pairs with each pair having the same citation count. The entropy weight method was used to weigh the three knowledge flow patterns to make a holistic evaluation on articles’ citation impact. Experiment results show that articles exhibit completely different citation impact, although they have the same citation count. In addition, articles will have similar citation impact even if they vary largely in the total citation count. The results show that there is a necessity to consider the knowledge flow patterns while analyzing articles’ citation impact.

In conclusion, detecting the citation impact from the perspective of knowledge flow will provide a novel consideration to evaluate the value of publications. Mainly, the focus has been on the evaluation of a publication’s accumulated influence gathered using a citation-count-based assessment. However, as discussed in this paper, citation activities should not be expressed solely as a single number, but they should reflect various citation properties from the perspective of knowledge flow. The citation performance of publications can vary according to different citation patterns, even if they have the same citation count. This can be helpful in evaluating publications and can be useful for decision-makers to evaluate the academic performance of different researchers or different institutions. It can also be valuable in steering research policy and for hiring or promotion decisions. In different applications, the three aspects and weights proposed in this paper are not invariable. Decision-makers can select or focus on different aspects of knowledge flow as required and formulate a reasonable weight system in line with the actual situation, to complete the evaluation task based on a realistic point of view rather than the number of citations.

It is worth mentioning that although the current work is to take journal papers as an example to discuss the issue of citation impact assessment of academic achievements, the ideas and methods of this work are still applicable to other types of academic achievements, such as conference papers. Taking conference papers as an example, the total number of citations is also the result of the accumulation of citations in the citation life. The citations obtained in different periods will also have different knowledge flow intensity, which will have different influence on their ability of attracting new citations in the future. In addition, through the occurrence of citation behavior, conference papers will also have an impact on other academic entities, resulting in different characteristics of knowledge diffusion intensity and knowledge transfer intensity. Therefore, there are also differences in the characteristics of knowledge flow in the citation behavior of conference papers. By using entropy weight method to synthesize the knowledge flow characteristics of conference papers, the comprehensive evaluation results of citation influence of conference papers can also be obtained.

Although some interesting phenomena have been found in this study, which has certain reference value for the construction of scientific and effective evaluation mechanism of academic papers, there are still some limitations. The method proposed in this paper can not be directly used to evaluate the influence of academic papers in different disciplines. There are great differences in citation characteristics in different disciplines, which leads to significant differences in the three-dimensional characteristics of knowledge flow. This method can not be directly used to distinguish the citation effect of articles in different disciplines before finding appropriate technology to effectively measure the differences brought by domain features and using these differences to complete the standardization of domain features. In the future research, we will further explore the standardized method of domain characteristics to solve the deviation of influence evaluation of academic papers in different fields.

## Supporting information

S1 File Article data and the accordingly citation data in Table 1. (XLSX)
